# Initial Characterization of Male Southern Stingray (*Hypanus americanus*) Reproductive Parameters and Preliminary Investigation of Sperm Cryopreservation

**DOI:** 10.3390/ani11092716

**Published:** 2021-09-17

**Authors:** James D. Gillis, Linda M. Penfold, Natalie D. Mylniczenko

**Affiliations:** 1 South-East Zoo Alliance for Reproduction & Conservation, 581705 White Oak Road, Yulee, FL 32097, USA; Linda.Penfold@sezarc.com; 2 Disney’s Animals, Science, and Environment, Disney’s Animal Kingdom, The Seas with Nemo and Friends, Walt Disney’s Parks and Resorts^®^, Bay Lake, FL 32830, USA; Natalie.Mylniczenko@disney.com

**Keywords:** sperm, cryopreservation, elasmobranch, semen, testosterone

## Abstract

**Simple Summary:**

Understanding the reproductive biology of a species is critical for the development of biobanks and assisted reproductive techniques to aid in the genetic management of isolated populations. Male southern stingray (*Hypanus americanus*) reproductive examinations were opportunistically conducted in March and June. Semen and plasma were collected to characterize ejaculate parameters and to investigate the effect of plasma total testosterone on semen quality. Semen was used for preliminary sperm cryopreservation studies. Changes in semen quality were observed with changes in plasma testosterone concentrations and body conditions. Southern stingray spermatozoa were highly sensitive to cooling rates with slower rates, producing a higher post-thaw survival.

**Abstract:**

This study investigated the reproductive biology and sperm cryopreservation of ex situ southern stingrays (*Hypanus americanus*) by semen collection and characterization and the development and validation of an enzyme-linked immunoassay for plasma total testosterone. Semen was collected in March and June using a manual massage technique, and the ejaculates were assessed for volume, pH, osmolarity, motility, status (0–5 scale: 0 = no forward progression, 5 = rapid linear progression) and total sperm count. Semen was extended in Hank’s elasmobranch ringer solution containing 10% DMSO, 10% glycerol or 5% glycerol with 5% N-methylformamide and cryopreserved using a conventional freezing method (~−50 °C/min) or a modified slow freezing method (~−3 °C/min). Body condition was scored from 1–5 and was noted to be low in March (1.93 ± 0.07) due to feeding practices and increased by June (2.93 ± 0.05) after dietary corrections were made. A concomitant increase (*p* < 0.05) in plasma total testosterone concentration and sperm motility was noted between March (8.0 ± 7.2 ng/mL, 5.71 ± 2.77%) and June (97.3 ± 11.3 ng/mL, 51.4 ± 14.3%). Samples cryopreserved using a modified slow freeze method (~−3 °C/min) had higher post-thaw motility and plasma membrane integrity than conventionally cryopreserved samples. Data indicate that southern stingray sperm morphometrics adheres to those of other elasmobranch species and that a slow cooling rate may be an avenue of research to improve southern stingray sperm survival during cryopreservation.

## 1. Introduction

It is estimated that 25% of elasmobranch species are under threat of extinction because they are long-lived, slow to reproduce and are subject to by-catch and overfishing [1]. The reproductive biology of elasmobranchs is unique and frequently driven by environmental factors [2,3], yet is poorly studied in comparison to terrestrial mammals. Understanding the reproductive biology of elasmobranchs is important to generate self-sustaining populations in aquariums as insurance against extinction threats and to understand potential anthropogenic influences in the wild for best conservation approaches. Additionally, development of sperm cryopreservation techniques provide methods to preserve genetic material for future population management using artificial insemination, a conservation tool that has proven useful in teleost and mammalian species [4,5].

Southern stingrays (*Hypanus americanus*) are a widely distributed coastal species that range from New Jersey to Florida (USA), throughout the Gulf of Mexico, Bahamas and the Antilles, to the northern coast of South America [6]. Although the population is dispersed over a large range, little is known about this species’ reproductive biology. Although certain studies allude to a regionally dependent seasonality [6], anecdotal accounts indicate southern stingrays are nonseasonal and reproduce throughout the year under managed care and in the wild.

Southern stingrays are popular in aquariums and are relatively easy to handle, facilitating health and reproductive examinations. Previous work has focused on the reproductive biology of female southern stingrays [7] and with the exception for copulatory behaviours [8], male reproductive characteristics in this species have not been described. Semen characteristics, including pH, osmolarity, sperm concentration and motility, have previously been described in freshwater stingray species [9,10], but otherwise little information is available on semen parameters of male ray species. Until recently, many elasmobranch studies have been conducted post-mortem, but it has been recognized that non-lethal sampling should replace these methods [11]. Semen collection, hormone analysis and ultrasound examinations on live specimens are becoming more prevalent for investigating the reproductive biology of elasmobranch species, including the southern stingray [7,12,13]. In addition, ultrasonography can be a useful tool in assessing body condition, an indicator of reproductive function in elasmobranch species [12]. As liver size is a classic measure of body condition in elasmobranchs [13], researchers can use ultrasound as a non-invasive technique to establish body condition [14].

To date, there are no well-established sperm cryopreservation protocols for elasmobranch species [15]. Historically, media used to investigate short-term cold storage and cryopreservation of spermatozoa have been formulated to be physiologically similar to elasmobranchs body fluids [16]. Previous sperm cryopreservation studies in elasmobranch species have yielded a post-thaw motility of ≤35%, but with limited duration of motility [16,17,18]. Species differences in tolerance to cryoprotectants was noted and recommendations were made for further research into the development of species specific techniques [16]. Species specific cryopreservation methods are warranted if genetic material, such as sperm, is to be stored in biobanks. In another study, attempts to cryopreserve sperm from whitespotted bamboo sharks (*Chiloscyllium plagiosum*) proved unsuccessful (Penfold, personal observation) though led to the development of short-term cold (5 °C) storage protocol that proved effective for shipping semen between institutions for artificial insemination [19].

Seasonal increases in testosterone, testicular size and spermatogenesis have been observed across diverse taxa [20,21,22]. In elasmobranchs, seasonal plasma testosterone concentrations have been shown to correlate directly with semen quality in sand tiger sharks (*Carcharias taurus*), which in turn were correlated with water temperature, thus plasma testosterone can be a useful indicator of season and semen quality [3]. Testosterone and its relation to semen quality has not yet been measured in male southern stingrays and appropriate assay validation is important for reliable results. Though testosterone is highly conserved across species, differences in how the hormone is transported throughout the body may vary. Previous work has demonstrated that reproductive hormones have a high affinity to elasmobranch steroid binding globulins requiring pre-treatment to minimize plasma matrix effects before analysis [7].

The objectives for this study were to: (1) characterize the ejaculate of southern stingrays, (2) understand the relationship between plasma total testosterone and semen quality and (3) investigate the effects of different cryoprotecting agents and freezing methods on the post-thaw survival of sperm as a preliminary step in developing long-term storage protocols.

## 2. Materials and Methods

### 2.1. Animals

This study was approved by Disney’s Animal Care and Welfare Committee. Male southern stingrays (*n* = 7), ranging from 10 to 13 years of age were housed in 12,000-gallon rectangular enclosures at Florida Marine Aquaculture (Ruskin, FL, USA) with constant temperature and a natural photoperiod. Water salinity was 28 to 30 ppt (875 to 925 mOsm).

Animals were fed capelin, silversides and shrimp and were supplemented with 0.5 g/day of Mazuri^®^ Shark and Ray Supplement (5MD8, 1.5 g tablet, Mazuri Exotic Animal Nutrition, St. Louis, MO, USA). Animals with low body condition scores were identified in March during routine examinations. Feeding practices were altered to improve consumption, including increased volume of food, and target and focus feeding versus free/group feeding. Caloric intake was increased by altered distribution of proportions to favour increased calories; a second health examination in June was conducted to evaluate the effects of these changes.

Blood samples were collected from males using a 20-gauge needle and 3 mL of blood was collected from a radial wing vessel by inserting the needle perpendicularly to the skin between ceratotrichia approximately one-fourth of the fin width from the lateral edge of the coelomic cavity. Blood was transferred to lithium heparinized tubes that were inverted multiple times and centrifuged at 1300× *g* for 10 min. Plasma was transferred into 2 mL cryovials (Simport Scientific Inc., Saint-Mathieu-de-Beloeil, QC, Canada) and stored at −80 °C until hormone analysis.

### 2.2. Body Condition Assessment

**Figure 1:**
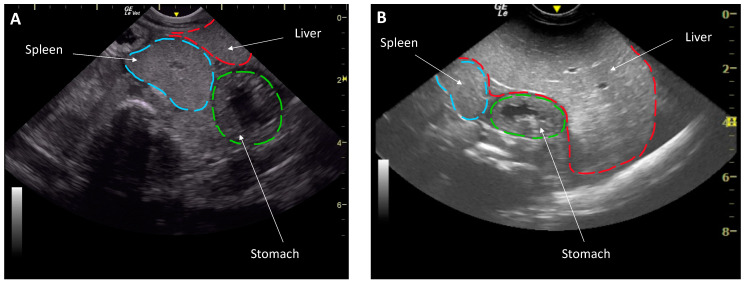
Liver assessment by ultrasonography in a dorsally recumbent male southern stingray (*Hypanus americanus*) of a low body condition in March with a smaller liver (**A**) and an animal in good body condition June (**B**) with a larger liver. Note the depth of the organs illustrated by the gridlines in cm on the right.

The change in body condition between health examinations offered a unique opportunity to evaluate the effect of body condition on reproductive function. Body condition was subjectively evaluated based on the appearance of the celomic fill and ultrasound characteristics [14]. Then a body condition score was assigned on a scale of 1 to 5 (1 being emaciated, 3 normal and 5 being morbidly obese) by the same researcher (N.D.M.) to eliminate inter-assessor variability. Coelomic ultrasound was performed transcutaneously in dorsal recumbency using a GE LogiqE with a 5–8 MHz curvilinear and 5–10 MHz linear transducer (General Electric Company, Boston, MA, USA) [14]. An animal of a low body condition had an ultrasound characterization that showed a small liver that spanned <50% of the coelom, had the spleen and liver thickness similar to each other and the liver and spleen were isoechogenic. Conversely, a good body conditioned animal had a liver >50% of the coelomic length, a liver that was thicker than the spleen as well as hyperechoic to the spleen (Figure 1). Average depth of the spleen in good condition was at 4 cm and <2 cm in low condition.

Weight was determined by taring a scale with a shallow tub before the animal was transferred into the tub, rapidly weighed and returned to the water, the tub was then reweighed to subtract any additional water that may have inadvertently been transferred.

### 2.3. Semen Collection, Assessments and Cryopreservation

#### 2.3.1. Media Preparation

All media and diluents were prepared with cell culture grade reagents from Millipore Sigma, St. Louis, MO, USA. Artificial seawater (ASW) was prepared by adding 40 g of sea salts (S9883, Millipore Sigma, St. Louis, MO, USA) to 1 L ultrapure water and adjusting to 1050 ± 10 mOsm. Hank’s elasmobranch ringer solution (ER) was prepared with 280 mM NaCl, 6 mM KCl, 5 mM CaCl_2_-2H_2_O, 4.6 mM MgCl_2_-6 H_2_O; 0.5 mM Na_2_SO_4_, 0.5 mM NaH_2_PO_4_-2H_2_O, 8 mM NaHCO_3_, 350 mM urea, 106 mM trimethylamine N-oxide, 0.9 mM glucose and pH 7.5, 1050 ± 10 mOsm [17]. Media and diluents were prepared using a sterile technique with ultrapure water and were successively filtered through 0.45 μm and 0.22 μm syringe filters (Santa Cruz Biotechnology, Dallas, TX, USA).

#### 2.3.2. Semen Collection and Analysis

Males were anesthetized with 70 mg/L tricaine methane sulfonate (Syndel, Ferndale, WA, USA) buffered 1:1 with NaHCO_3_ for routine health examinations (physical examination with ultrasound, blood sampling and analysis, and semen collection) in March and June.

The cloaca was rinsed with sterile 0.9% saline (Baxter Healthcare, Deerfield, IL, USA), the biofilm gently removed with soft gauze, rinsed again and dried to avoid contamination of the semen with seawater. Semen was collected by gently massaging the area lateral to the urogenital pore, which usually resulted in immediate release of semen into the cloaca. White/cream coloured semen was generally collected within 30 s and collected with a Pasteur pipette (Karter Scientific, Lake Charles, LA, USA) and transferred into 1.8 mL microcentrifuge tubes (Santa Cruz Biotechnology, Dallas, TX, USA). Ejaculates grossly contaminated with urine or fluid from the valvular intestine, characterized either by a large volume of liquid filling the cloaca or by brown/green discharge, were not used in the study. Samples were maintained at 20 °C and protected from light until processing.

Ejaculates (*n* = 14, from 7 males (collected in March, June)) were assessed for volume, pH, osmolarity, concentration of spermatozoa, total motility, status (defined as linear progression on a scale of 0 to 5; 0 = no forward progression, 5 = rapid linear progression) [23,24] and plasma membrane integrity. Semen volume was measured with adjustable Gilson micropipettes (Gilson Incorporated, Middleton, WI, USA) and the pH was assessed using pH indicator strips (EM Science, Gibbstown, NJ, USA). Osmolarity was assessed using a freeze point depression osmometer (The Fiske^®^ Micro-Osmometer model 210, Norwood, MA, USA). The concentration of sperm was assessed with a haemocytometer (Daigger Scientific, Inc., Hills, IL, USA) by diluting semen 1:400 in double distilled water and counting sperm on both sides (10 µL volume/side; 4 squares of 16 on the four corners of the 25 squares in the middle) and taking the average. Total sperm count was determined by multiplying the sperm concentration by the ejaculate volume. Total motility (%) and status was subjectively determined using phase contrast microscopy (×40, Olympus B-Max 60 Microscope, Olympus Optical Co. Ltd., Tokyo, Japan) with a minimum of three separate fields examined at room temperature (23 °C). Samples were assessed raw and immediately following dilution of 25 µL of semen with 25 µL ASW in a 0.8 mL microcentrifuge tube (Daigger Scientific, Inc., Hills, IL, USA) and placing 3 µL on a microscope and covering with a 22 mm × 22 mm cover slip.

Images for spermatozoa measurements were obtained by placing 5 µL of ejaculate on a glass slide with 22 mm × 22 mm coverslip. Phase contrast microscopy (40×), an Olympus B-Max 60 and mounted Nikon digital camera (DS-2Mv, Nikon Corporation, Tokyo, Japan) was used to take micrographs of individual spermatozoa (*n* = 30) from each of two males. The length of the head, midpiece and flagellum were measured using Fiji (ImageJ) software package [25] by a single researcher to eliminate inter-assessor variability [26].

Methods to assess acrosomal presence were modified from previous established protocols in shark species [15,19]. In brief, the raw ejaculate was diluted 1:100 with ER and a 10 μL wet mount was evenly spread across a glass slide and allowed to air dry. Air-dried slides were (1) left unfixed or (2) fixed in methanol for 30 s. After the slides were dried, they were flooded with either (1) fluorescein-conjugated *Arachis hypogeal* (peanut) agglutinin (FITC-PNA, working solution of 1 mg/mL or 4 mg/mL, Thermo Fisher Scientific, Hamdon, NH, USA) or (2) fluorescein-conjugated *pisum sativum* agglutinin (FITC-PSA, working solution of 1 mg/mL or 4 mg/mL, Thermo Fisher Scientific, Hamdon, NH, USA) and incubated in a dark humidified chamber for 15 min at room temperature (23 °C). The slides were rinsed of excess stain and wet mounts were examined using an Olympus B-Max 60 epifluorescent microscope with filter cube U-M51005 for dual wavelength excitation at a magnification of ×40 − ×1000. In addition, the use of eosin-nigrosin staining [27] and Diff-Quick [28] to visualize acrosome-like structures were investigated.

**Figure 2:**
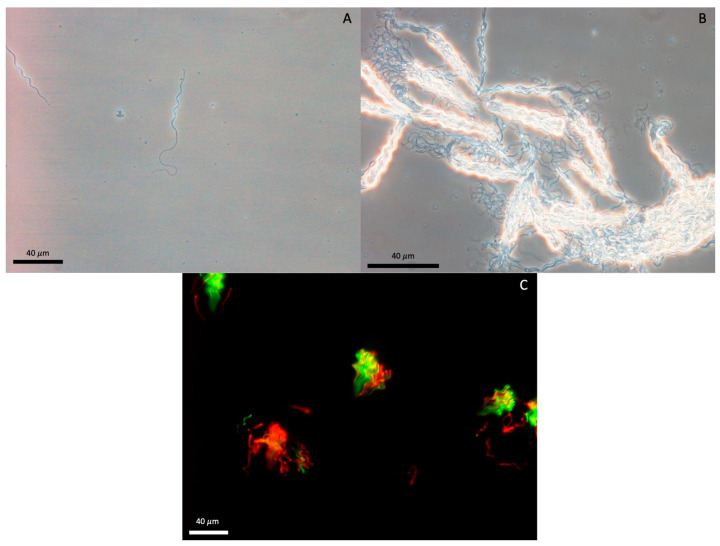
Southern stingray (*Hypanus americanus*) sperm (**A**) composed of a helical head, midpiece and flagellum. Spermatozeugmata (**B**) characterized by organized/aligned sperm bundles. Nuclei (**C**) of southern stingray sperm and spermatozeugmata intact (green) and disrupted (red) plasma membranes.

Plasma membrane integrity (PMI) was evaluated using a LIVE/DEAD sperm viability kit [29] (#L-7011; Molecular Probes, Inc., Eugene, OR, USA) comprised fluorescent stains Sybr-14 and propidium iodide (PI), previously validated in other elasmobranch species [3,17]. Diluted sperm were incubated at room temp for 10 min with 200 nM Sybr-14 and 24 μM of PI before examining using an Olympus B-Max 60 epifluorescent microscope with filter cube U-M51005 for dual wavelength excitation at a magnification of ×40. Spermatozoa fluorescing green over the head region were assessed as plasma membrane intact, and sperm fluorescing partially red or red over the head region were assessed as plasma membrane damaged (Figure 2C). A minimum of three fields were subjectively assessed and 100 sperm were assessed to generate a percent PMI.

#### 2.3.3. Sperm Cryopreservation

A modified ER previously used for elasmobranch sperm cryopreservation [17] was made by adding 20% (*v*:*v*) egg yolk to ER. The modified ER diluent was clarified by centrifugating at 5000× *g* for 90 min with the supernatant recovered and the pellet discarded. The resulting diluent was ER Fraction A. To prepare Fraction B the ER diluent was prepared as described above, with the addition of one of three cryoprotecting agent (CPA) treatments, selected based on previous published success in aquatic species [17,30]—(1) 20% glycerol (GLY), (2) 20% dimethyl sulfoxide (DMSO) or (3) 10% Gly with 10% N-methylformamide (GLY/MF; *v*:*v*; ER-GLY, ER-DMSO and ER-GLY/MF, respectively). Dilution of Fraction A with Fraction B 1:1 (*v*:*v*) yielded final CPA concentrations of 10% Gly, 10% DMSO or 5% Gly/5% MF.

To investigate effects of CPA on southern stingray sperm cryopreservation, samples from both March (*n* = 1) and June (*n* = 3) with a pre-freeze motility ≥50% were used as ~50% can be expected to be lost during the cryopreservation process [31] and good quality semen samples allow a fair assessment of the different CPA and cryopreservation methods. (1) First, semen samples were split into equal volumes and diluted to a concentration of ~200 × 10^6^ sperm/mL in ER Fraction A diluent. Conventional cryopreservation methods slowly cool sperm to 5 °C at a rate of −0.2 °C/min to avoid damaging the plasma membrane during a phase transition observed at ~15 °C in several terrestrial mammal species [32,33]. This approach was followed for the initial cooling phase to 5 °C. (2) Then, a volume of 250 µl extended semen was placed into a microcentrifuge tube and placed into a beaker of water containing 100 mL water at room temperature (23 °C) before transferring to a refrigerator to cool to 5 °C at a rate of ~−0.2 °C/min for 1 h. (3) When the samples reached 5 °C, the solutions were diluted in seven steps to reduce large osmotic cell excursions [34] to a 1:1 (*v*:*v*) dilution with either ER-GLY, ER-DMSO or ER-GLY/MF to a final concentration of ~100 × 10^6^ sperm/mL. Extended semen was assessed for motility and status at 5 °C 10 min after the addition of the Fraction B Solutions (ER-GLY, ER-DMSO or ER-GLY/MF). (4) Next, samples were equilibrated for 10 min at 5 °C before being loaded into 0.25 mL straws (Agtech Inc., Madison, KS, USA) and heat sealed. (5) Finally, straws were either cryopreserved using a conventional method or a modified slow freeze method. Conventionally, cryopreserved straws were placed 5.0 cm over liquid nitrogen (LN_2_; cooling rate of ~−50 °C /min) for 10 min before being submerged and stored in LN_2_. The slow freeze method involved transferring the straws from the refrigerator at 5 °C to a freezer (−20 °C), cooling at a rate of ~−3 °C/min to −20 °C, and then transferring the straws to an ultralow freezer cooling at a rate of ~−6 °C/min to −80 °C before being plunged and stored in LN_2_. All treatments were frozen in duplicate and stored at −196 °C for at least 24 h before being thawed.

#### 2.3.4. Post-Thaw Analysis

Duplicate straws were thawed in a 20 °C water bath for 10 s [17], wiped dry, and the contents released into a 1.8 mL microcentrifuge tube containing 0.5 mL of ER at 23 °C. Motility, status and PMI was evaluated every 10 min for the first half hour and then hourly for 4 h post-thaw. Total motility, status and PMI were recorded as described above.

### 2.4. Total Testosterone Enzyme-Linked Immunoassay

Plasma was analysed for total testosterone by enzyme-linked immunoassay (EIA) using a polyclonal anti-testosterone-6-carboxymethyl oxime antiserum (R156/7; Munro, University of California, Davis, CA, USA) and testosterone conjugate (testosterone-3-carboxymethyl oxime:horseradish peroxidase), previously validated in female southern stingrays [7]. Stingray plasma was treated 1:1 with dissociation reagent (cat #X017-5ML; Arbor Assays, Ann Arbor, MI, USA) in EIA buffer (0.04 M NaH_2_PO_4_, 0.06 M Na_2_HPO_4_, 0.15 M NaCl, 0.1% BSA, pH 7.0). Parallelism between serial dilutions of sample (*n* = 7, 1:5–1:640) and the standard curve were determined by both visual inspection and t-test for differences of slope (*p* > 0.879). Recovery of known amounts of testosterone (standard concentrations 0.04–5.0 ng/mL) added to pools of diluted plasma was 98.7% (y = 1.0073x + 0.0035, r^2^ = 0.998). Treated plasma was run in duplicate after diluting 1:80 for assay on a single plate. Testosterone assay sensitivity was 0.08 ng/mL. Mean ± SD intra-assay coefficient of variation was 2.49% ± 0.38. Dissociation reagent alone (background) yielded 8.0 ng/mL and was subtracted from results.

### 2.5. Statistical Analysis

Statistical analyses were performed using GenStat 19th edition (VSN International, Hemel Hempsted, UK). Non-parametric data for ejaculate characteristics and testosterone concentration by month was analysed using Wilcoxon signed rank test. The Kruskal–Wallis test was used to evaluate differences in the pre-freeze sperm motility in various CPA. However, due to limited sample size in each cryopreservation method (conventional vs. modified slow freeze), statistical analysis assessing outcome differences was not possible and descriptive statistics were used for post-thaw sperm parameters. An Alpha value of *p* < 0.05 was considered significant. Data are presented as Mean ± SEM.

## 3. Results

### 3.1. Body Condition

Ultrasonography showed that all individuals had livers described as small in March and large in June, taking up ≥50% of the coelom (Figure 1). In March, increased nutrition was immediately offered and the average stingray weight in March increased by ~15%, from 6.85 ± 0.32 kg to 7.22 ± 0.28 kg in June. The body condition score similarly increased from 1.93 ± 0.07 in March to 2.93 ± 0.05 in June.

### 3.2. Semen and Sperm Characteristics

Semen and sperm characteristics are shown in Table 1. The spermatozoa were filiform, with a helical head (38.3 ± 0.18 μm) comprised ~4.3 gyres (6.1 if midpiece is included), midpiece (14.5 ± 0.16 μm) and flagellum (64.1 ± 0.44 μm; Figure 2A). Ejaculates consisted of a mixture of free sperm distributed throughout the accessory fluid, and spermatozeugmata (Figure 2B) characterized by aligned sperm bundles [35].

Attempts to visualize an acrosome using previously published and modified protocols, including eosin-nigrosin stain and Diff-Quick were unsuccessful. The majority of the spermatozoa did not show any signs of fluorescence over the proximal end of the sperm head. On rare occasion, slight mottling over the proximal region was observed; however, it was unclear whether this was associated with potential release of acrosome contents or remnants of the matrix from the spermatozeugmata.

All ejaculates collected contained spermatozoa, and 42.8% of ejaculates in March and 85.7% in June contained motile sperm. Average sperm motility in the raw ejaculate increased (*p* < 0.05) from 5.7 ± 2.8% in March to 51.4 ± 14.3% in June. The motility of the spermatozoa adhered to a ubiquitous sperm locomotion strategy observed across taxa, which is helical with three-dimensional flagellar beating [36,37]. Southern stingray sperm did not require the addition of ASW to initiate motility (Table 1). Further, motility and PMI declined within 1 h after dilution with ASW compared to the raw ejaculate.

An increase (*p* < 0.05) in semen volume and decrease (*p* < 0.05) in concentration between March and June resulted in no change (*p* > 0.05) in total sperm count (Table 1).

### 3.3. Sperm Cryopreservation

**Figure 3:**
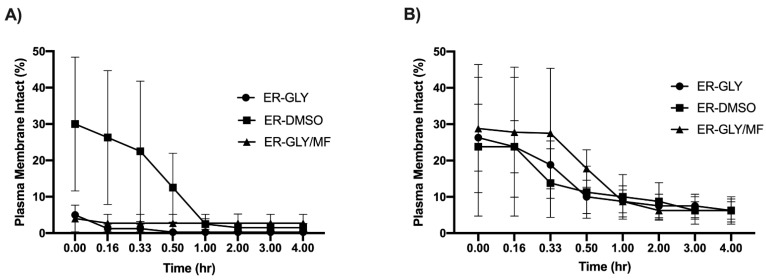
Plasma membrane integrity ($\bar{x}$ ± SEM) of frozen-thawed southern stingray (*Hypanus americanus*) sperm (March *n* = 1, June *n* = 3) in using (**A**) conventional and (**B**) modified slow freeze methods. ER = Hank’s elasmobranch ringer solution; GLY = glycerol; DMSO = dimethyl sulfoxide; MF = N-methylformamide.

The addition of CPAs before cryopreservation had no effect (*p* > 0.05) on sperm motility (Table 2). A modified slow freezing method gave consistently higher post-thaw sperm motility than conventional freezing (Table 3). Immediately after thawing (0 h), highest post-thaw motility (16 ± 7.5%) was obtained for sperm cryopreserved in GLY and frozen using the modified slow rate, though after 1 h, motility had dropped to 4 ± 2.27%. Even though highest numbers of plasma membrane intact sperm (30 ± 18%) were initially obtained with DMSO and conventional freezing, more sperm retained plasma membrane integrity for a longer period of time following a modified slow freezing (Figure 3).

Variations in individual ejaculate ability to withstand the cryopreservation process were noted and post-thaw motility ranged from 0–25% and PMI from 0–80% (Table 3), demonstrating the usefulness of selecting good quality ejaculates for cryopreservation trials. Motility and PMI were correlated (r^2^ ≥ 0.86) in all treatment groups except for samples conventionally cryopreserved in 10% DMSO (r^2^ = 0.21).

### 3.4. Testosterone

In the month of March, mean plasma total testosterone concentration was 8.0 ± 7.2 ng/mL. Six of seven males had plasma total testosterone concentrations below the assay sensitivity of 0.08 ng/mL, with one male’s plasma total testosterone concentration at 51.2 ng/mL. Higher concentrations (*p* < 0.05) of testosterone were measured in all seven males in June, with an average concentration of 97.3 ± 11.3 ng/mL (Table 1).

## 4. Discussion

Samples cryopreserved using a modified slow freeze method produced higher post-thaw survival parameters than conventionally cryopreserved samples (Table 3). The highest post-thaw motility was achieved using the modified slow freeze method with 10% GLY (*v*:*v*). The post-thaw motility was initially 16.3 ± 7.47% but declined to 4.0 ± 2.27% after ~1 h. For one individual, the post-thaw motility of 35% after thawing and 25% after 1 h was promising and indicates that further refinement of the modified slow freeze protocol and improved pre-freeze sperm quality could yield better results. These findings are in agreement with previous findings in other batoid species [17,18]. Higher proportions of intact plasma membranes than motile sperm were observed post-thaw, suggesting that the plasma membrane over the flagellum is possibly more sensitive to cryopreservation events than the plasma membrane over the head region, as has been demonstrated in mammalian species [34]. Further research is warranted to continue to attempt to develop elasmobranch sperm cryopreservation protocols, but concomitant studies investigating cold storage also will be useful for short-term storage of elasmobranch sperm that can similarly be used to support managed breeding programs to preserve genetic diversity.

The ability to reliably collect semen by manual massage technique from southern stingrays continues to support non-lethal methods of sampling in elasmobranchs. In general, the differences between semen and sperm parameters between March and June were minimal except for concentration, volume and motility. Lack of accessory fluids were the primary reason for higher sperm concentration but not total sperm count. The slight changes in pH and osmolarity between March (7.43 ± 0.27; 776 ± 66.9 mosm) and June (8.06 ± 0.04, 857 ± 38.9 mosm) would explain the significant differences in motility observed between months [38,39]. The forces driving these changes in semen characteristics (osmolarity and pH) remain ambiguous as (1) a change in body condition (from low to good) was observed and (2) potential seasonal effects on semen quality have not been studied in southern stingrays. Either of which could be causing a change in semen quality. Longitudinal analysis of ejaculate characteristics over the course of a year is required to fully understand the effect of season on southern stingray ejaculate quality.

Semen collected from sand tiger sharks and bamboo sharks required activation of motility through dilution with ASW [3,19], which was in contrast to southern stingray sperm which did not require the addition of ASW to initiate motility (Table 1). Furthermore, motility and PMI declined within 1 h after dilution with ASW compared to the raw ejaculate. Although the present study did not investigate the effect of osmolarity, it is likely that the ASW and ER diluents, which were a higher osmolarity than the raw ejaculate, could be impacting sperm motility parameters. Further investigations into osmotic tolerance are warranted to better understand the effect of osmolarity on sperm motility and survival.

The spermatozoa were structurally similar to other elasmobranch species and displayed the characteristic filiform shape with a helical head. However, the inability to visualize an acrosome using PNA/PSA, eosin-nigrosin stain or Diff-Quick warrants further investigation. In general, nonmammalian acrosomes contain less carbohydrates than mammalian species [40], which may explain why PNA/PSA, which bind subunits of carbohydrates, were ineffective in identifying an acrosome structure in southern stingray sperm. Utilization of transmission electron microscopy could elucidate the presence or absence of an acrosome structure in southern stingray sperm.

Phylogenetic studies have demonstrated that the structure and function of testosterone is highly conserved across species [41]. Spermatogenesis, motility and several other reproductive functions, have been correlated to seasonal testosterone production in several terrestrial species, as well as wild sand tiger sharks, Atlantic stingrays (*Dasyatis sabina*) and round stingrays (*Urobatis halleri*) [3,42,43,44]. As it pertains to the findings of this study, the observed increase in plasma testosterone concentrations from March to June was coupled with an increase in total sperm motility in the raw ejaculate, more ejaculates containing motile sperm, improvement of body condition, increased liver size and weight gain. It remains difficult to separate potential seasonal changes from changes in body condition. Although plasma testosterone was significantly higher in June, body condition again cannot be ruled out as contributors to the difference, especially as cholesterol is the primary component of steroid hormone synthesis and also has previously been correlated with body condition in elasmobranchs [45]. Conversely, further research will determine whether season has any effect on southern stingray ejaculates. Nonetheless, for plasma testosterone, whether a function of season and/or change in body condition, coupled with the fact that plasma testosterone concentrations in female southern stingrays have been reported to be lower (<10 ng/mL) [7] than male samples collected in June in this study, demonstrates a biological validation for the testosterone assay in the present study. Additional work in elasmobranchs has shown that alternative pre-treatments for serum may yield less background and more sensitive results [46,47], suggesting a future area for optimization of testosterone measurements with this assay.

## 5. Conclusions

In this first assessment of southern stingray semen, results show that the sperm structure is similar to other elasmobranchs studied thus far and that the sperm did not require activation of motility. Validation of a total testosterone EIA for this species demonstrated that higher testosterone was associated with increased body condition and sperm motility, though longitudinal analysis of plasma testosterone and semen will be important to fully characterize the reproductive seasonality of male southern stingrays.

Southern stingray sperm appear to be highly sensitive to the cryopreservation process, which warrants further investigations into the identification of optimal cooling rates and cryoprotecting agents. In the meantime, the development of short-term storage methods may be prudent to support artificial insemination strategies of managing stingray populations under managed care to support conservation goals.

## Figures and Tables

**Table 1:** Southern stingray (*Hypanus americaus*) ejaculate characteristics and plasma testosterone concentrations ($\bar{x}$ ± SEM) by month.

Parameter	March *n* = 7	June *n* = 7
Volume (mL)	0.29 ± 0.1 ^a^	2.12 ± 0.45 ^b^
pH	7.43 ±0.27	8.06 ± 0.04
Osmolarity (mOsm)	776 ± 66.9	857 ± 38.9
Total motility; raw (%)	5.71 ± 2.77 ^a^	51.4 ± 14.3 ^b^
Status; raw (0–5)	0.71 ± 0.36	1.5 ± 0.46
Total motility; in ASW (%)	32.9 ± 12.0	33.6 ± 15.5
Status; in ASW (0–5)	1.21 ± 0.46	1.57 ± 0.57
Concentration (×10^6^ sperm/mL)	1296 ± 353 ^a^	371 ± 104 ^b^
Total sperm count (×10^6^)	280 ± 88.4	284 ± 46.2
Plasma total testosterone (ng/mL)	8.0 ± 7.2 ^a^	97.3 ± 11.3 ^b^

Superscript letters (a, b) indicate significant differences in sperm parameters between months. ASW = artificial sea water.

**Table 2:** Southern stingray (*Hypanus americanus*) (June *n* = 3) sperm motility parameters at 5 °C extend in various cryoprotecting agents ($\bar{x}$ ± SEM).

Extender	Total Motility (%)	Status (0–5)
ER Fraction A	43.3 ± 17.6	2.17 ± 0.83
ER-GLY	36.7 ± 17.6	1.83 ± 0.72
ER-DMSO	23.3 ± 13.3	2.0 ± 0.76
ER-GLY/MF	36.7 ± 17.6	1.83 ± 0.73

ER = Hank’s elasmobranch ringer solution; GLY = glycerol; DMSO = dimethyl sulfoxide; MF = N-methylformamide.

**Table 3:** Longitudinal post-thaw survival of southern stingray (*Hypanus americana*) sperm cryopreserved using various freezing methods and cryoprotecting agents ($\bar{x}$ ± SEM, Range).

		Cryoprotecting Agent					
		GLY		DMSO		GLY/MF	
	Time (h)	Motility (%)	PMI (%)	Motility (%)	PMI (%)	Motility (%)	PMI (%)
Modified slow freeze	0	16.3 ± 7.47 0–35	26.3 ± 9.21 5–50	9 ± 8.67 0–35	23.8 ± 19.1 0–80	6.25 ± 3.75 0–15	28.8 ± 17.6 0–80
	1	4 ± 2.27 0–10	8.75 ± 4.27 0–20	6.5 ± 6.17 0–25	10 ± 6.12 0–25	1.5 ± 1.19 0–5	8.75 ± 3.15 0–15
	4	2.5 ± 1.44 0–5	6.25 ± 3.15 0–15	2.5 ± 2.5 0–10	6.25 ± 3.75 0–15	1.5 ± 1.19 0–5	6.25 ± 2.39 0–10
Conventional cryopreservation	0	1.5 ± 1.19 0–5	5 ± 3.34 0–15	6.75 ± 6.09 0–25	30 ± 18.4 0–80	1.5 ± 1.19 0–5	4 ± 3.67 0–15
	1	0.25 ± 0.25 0–1	0.25 ± 0.25 0–1	1.25 ± 1.25 0–5	2.5 ± 1.44 0–5	1.5 ± 1.19 0–5	2.75 ± 2.43 0–10
	4	0.25 ± 0.25 0–1	0.25 ± 0.25 0–1	0 0	1.5 ±1.19 0–5	0.25 ± 0.25 0–1	2.75 ± 2.43 0–10

PMI = plasma membrane integrity; GLY = glycerol; DMSO = dimethyl sulfoxide; MF = N-methylformamide.

## Data Availability

The data presented in the present study are available upon request from the corresponding author.
